# Cytokine profiles of filarial granulomas in jirds infected with *Brugia pahangi*

**DOI:** 10.1186/1475-2883-5-3

**Published:** 2006-03-16

**Authors:** Ramakrishna U Rao, Thomas R Klei

**Affiliations:** 1 Infectious Diseases Division, Internal Medicine Department, Washington University School of Medicine, Campus Box 8051, St. Louis, MO 63110, USA; 2 Department of Pathobiological Sciences, School of Veterinary Medicine, Louisiana State University, Baton Rouge, LA 70803, USA

## Abstract

**Background:**

A granulomatous inflammatory response develops in jirds infected subcutaneously or intraperitoneally with filarial nematodes namely *Brugia pahangi *and *B. malayi*. Previous studies by light and electron microscopy have shown cellular inflammatory responses in and around these granulomas. Furthermore, the cellular inflammatory responses of granulomas found in the lymphatics and peritoneal cavity appear to be similar. The purpose of this study was to determine the cytokine profiles of granulomas in the peritoneal cavity of *B. pahangi*-infected jirds and to determine whether the granulomas release any proinflammatory cytokines *ex vivo*.

**Methods:**

A semiautomated quantitative polymerase chain reaction (Q-PCR) was performed on cDNA prepared from the granulomas of infected jirds to study the species-specific mRNA expression of IL-2, IL-4, IFN-γ, IL-5, and IL-10. Genomic DNA was extracted from the granulomas, and parasite DNA was detected by Q-PCR by amplifying the *Hha*I repeat sequence. The levels of the inflammation-causing cytokines IL-6 and TNFα that were secreted by the granulomas were measured by cell-based assays.

**Results:**

Florid granulomas showed higher levels of IFN-γ than other cytokines, linking this Th1 cytokine to the granulomatous inflammation that develops in jirds and humans. IL-4 expression was much lower than that of IFN-γ but higher than that of IL-10. A low level of IL-5 mRNA expression was detectable in all granulomas as was the level of IL-2 expression. The levels of the inflammatory cytokines IL-6 and TNFα, secreted by intact granulomas, spontaneously increased by 48 h after culture. Parasite antigen stimulation and subsequent release of IL-6 and TNFα by the granulomas indicated a moderate increase in the levels of these two cytokines. The amplification of the *Brugia Hha*I repeat DNA and *Wolbachia *16S rDNA indicated worm components and bacterial components in the granulomatous tissue.

**Conclusion:**

Granuloma development in filarial infections is a complex process involving cellular reactions responding to parasite/bacteria and their components. The interactions between worm-derived granulomas and their hosts are dynamic and multifaceted. The data collected thus far suggest that the expression profiles of many of the measured cytokines in the lymphoid tissues of *Brugia*-infected jirds are different from those of the cytokines in granulomas. Moreover, granulomas have the ability to secrete the inflammatory cytokines IL-6 and TNFα.

## Background

Lymphatic filariasis, caused by the filarial nematodes *Wuchereria bancrofti*, *Brugia malayi*, and *Brugia timori*, affects more than 120 million people worldwide. The most common clinical signs of infection are recurrent episodes of filarial fever coupled with inflammation of the affected lymphatics [1-4]; in most cases, these symptoms are accompanied by the appearance of microfilariae in the peripheral blood [3,5,6]. In lymphatic filariasis, the pathological lesions are found in the lymphatics as a result of their dysfunction and the attendant injury to the walls and valves [7-10]. The lesions are generally granulomas with deposits of collagenous material. In humans, granulomatous lesions around nodules with a filarial aetiology have been observed [11,12]. In endemic areas, lumps in the affected breasts and testicles and a coin-like shadow in the lungs have been attributed to granulomatous reactions to filarial worms. Similarly, lymphatic lesions in animals infected with filarids are primarily granulomatous [13-18]. The origin of granulomatous inflammation in lymphatics is commonly associated with the host's response to dead or dying worms and to their somatic excretions and secretions. Interestingly, it has been shown that the granulomatous lesions resulting from mycobacteria and schistosoma are mediated by T cells [19,20].

Among rodents, jirds are highly permissive for filarial infections, including *Brugia *spp., *Litomosoides sigmodontis*, and *Acanthocheilonema viteae*. Previous studies have shown that a granulomatous inflammatory response develops in *Brugia*-infected jirds and consists of different types of granulomatous lesions involving several cell types. However, the lesions in the lymphatics and the peritoneal cavity are similar [21,22].

We have observed that the Mongolian jird-*Brugia *experimental model reflects, in many ways, the status of humans infected with *B. malayi *or *W. bancrofti *[23,24]. In particular, initially, infection with the filarial nematode *Brugia *in jirds produces a cellular hyperresponsiveness to worm antigen around 28 days after infection (DAI), and the number of lymph thrombi (LT) is increased after 56–90 DAI. During this period of infection, jirds also show an increase in the number and size of LT and an increased pulmonary granulomatous (PGRN) response to *Brugia *antigen-coated sepharose beads embedded in their lungs [23,24]. After approximately 90 DAI, the time at which microfilaremias are well established in the circulation, the cellular response to filarial antigens decrease, as do the numbers and sizes of LT and PGRN inflammation [23,24]. In contrast, the LT that are formed in the lymphatics during the onset of a *Brugia *infection remain unresolved during the entire course of infection. The results from these studies suggest that multiple mechanisms are involved in the cell-mediated formation of PGRN and LT. However, much remains unknown about the specific reactions associated with the development of filarial granulomas in infected tissues and about granuloma-associated pathology in humans and animals.

Recently, many jird cytokine genes were characterized and highly sensitive and specific quantitative polymerase chain reaction (Q-PCR) methods were developed to study the cytokine expression in lymphoid and nonlymphoid tissues [25,26]. This paper reports on the major T-cell cytokine gene-expression profiles in granulomas in the peritoneal cavity of jirds following a primary infection induced by infective third-stage larvae (L3). Using the polymerase chain reaction, we found that these granulomas originated from parasites/bacteria and were responsible for the localized cellular inflammatory responses involving cytokines.

## Methods

### Animals and infections

Mongolian jirds (*Meriones unguiculatus*) were obtained from Charles River (Wilmington, MA) at 8 weeks of age. They were fed standard rodent chow and given water *ad libitum*. *B. pahangi *infective L3 were recovered from infected *Aedes aegypti *mosquitoes using Baermannization, as previously described [23], and randomly divided into aliquot doses of 100 larvae in 0.5 ml of RPMI (GIBCO/Invitrogen Corp., Carlsbad, CA). Jirds were injected intraperitoneally (i.p.) with 300 *B. pahangi *L3, and 300 DAI, necropsies were performed on six jirds infected i.p. to recover florid granulomas.

### Worm antigen

To prepare a soluble extract of worm antigen, adult worms were aseptically obtained from the peritoneal cavities of infected jirds and placed in phosphate-buffered saline (pH 7.2), as described previously [23]. The protein content in the adult worm extract was estimated by a Bradford assay [23] and aliquots of this soluble extract were stored at -70°C until used.

### Granulomas

**Figure 1:**
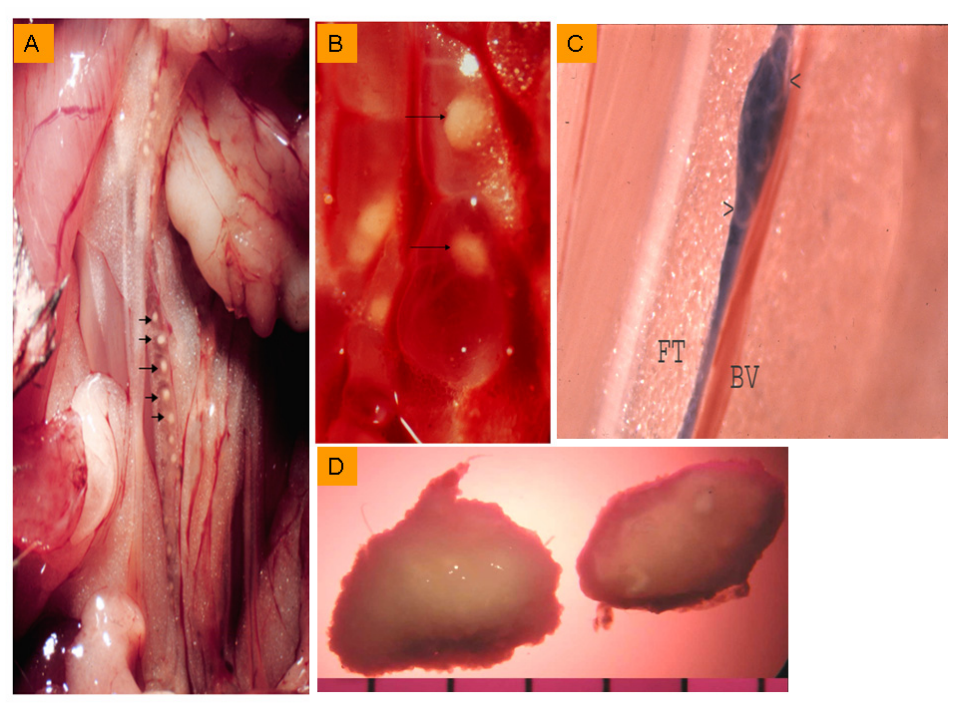
Pathological lesions in jirds infected with *Brugia pahangi*. A, Gross pathology of spermatic cord lymphatic vessel of a subcutaneously infected jird with *B. pahangi *(56 DAI) shows white granulomas (arrows) embedded in the vessel lumen (magnification 3×). B, Lymphatic vessel shows rounded and attached granulomas (arrows) (higher magnification, 7×). C, The same spermatic cord lymphatic vessel at the posterior end also contains a worm (arrows) tucked in the vessel lumen (lymphatic vessel was dyed with Evan's blue for contrast is adjacent to a blood vessel (BV) and embedded in fat tissue (FT), magnification 3×. D, Large nonadherent, florid granulomas from the peritoneal cavity of *B. pahangi*-infected jirds recovered at the chronic stage (300 DAI), magnification 3×.

Figure 1A and 1B shows adherent granulomatous lesions within the lymphatics of jirds infected subcutaneously with *B. pahangi*. In majority of animals, worms were visible within the lymphatics (Fig. 1C). Since the nonadherent florid granulomas (Fig. 1D) in the peritoneal cavity of chronically infected jirds are much larger (~5–10 mm) than the adherent ones, we selected these for study. All florid granulomas (Fig. 1D) were surgically removed from the peritoneal cavity of six infected jirds and washed three times with RPMI-1640 containing 100 U/ml penicillin, 100 μg/ml streptomycin, and 0.5 μg/ml gentamicin. Six granulomas were randomly selected from six jirds, and each granuloma was aseptically cut in half. One half of the granuloma was used for total RNA preparation, and the other half was used for genomic DNA extraction. Additional granulomas (n = 3) from these infected jirds were pooled and placed in cultures to study their secretion of IL-6 and TNFα cytokines *ex vivo*.

### Quantitation of cytokine mRNA

Extracts of granulomas were prepared in phosphate-buffered saline (n = 6, one half from each granuloma) by grinding the tissue to near homogeneity with a tabletop tissue grinder and then centrifuging for 10 min at 1000 rpm. The extract samples were resuspended in 0.5 ml of RNAStat 60 (Tel-test, Friendship, TX) and then snap frozen on dry ice. Total RNA was isolated from the sample by using chloroform extraction in accordance with the manufacturer's instructions. The quality and quantity of RNA was determined by a spectrophotometer (Beckman Coulter Inc., Fullerton, CA). Reverse transcription was carried out on 1 μg RNA, as previously described [27,28].

The jird T-cell cytokines interleukin (IL)-2 (Genbank accession no. X68779), IL-4 (L37779), IL-5 (L37780), IL-10 (L37781), interferon-γ (IFN-γ) (L37782), and the hypoxanthine phosphoribosyltransferase (HPRT) (L37778) were quantitated in all granuloma cDNA samples using the Q-PCR system 5000 (Applied Biosystems, Foster City, CA). Gene-specific oligonucleotide primers and probes were generated commercially (GeneLab, Baton Rouge, LA; Baron Biotech, Milford, CT). Positive strand primers were biotinylated on their 5' terminus. All probes were labeled with a chemiluminescent tris {2,2'-bipyridine} ruthenium (II) chelate (TBR; Baron Biotech) on their 5' terminus. Labeled primers and probes were purified by high-performance liquid chromatography to eliminate unlabeled oligonucleotides.

All PCR analyses were carried out in duplicate in 50-μl reactions, and PCR cycling conditions were as described before [27,28]. All values were normalized against those of the housekeeping gene, HPRT, as previously described [25]. Data are presented for each cytokine as the normalized luminosity unit ± standard error.

### Extraction and detection of parasite DNA

Total genomic DNA was extracted from *B. pahangi *adult worms (n = 5) and from the second halves of the 6 granulomas by using reagents supplied in the DNAeasy kit (Qiagen, Valencia, CA). Extracted genomic DNA was analysed for purity using a Beckman spectrophotometer and stored at -20°C until used. Parasite DNA detection was performed in genomic DNA preparations of granulomas by using the Q-PCR system 5000 (Applied Biosystems). For this purpose, *Hha*I repeat DNA sequence was amplified in a Q-PCR machine as described before [27]. The mean luminosity units obtained by the *Hha*I PCR assay served as a positive signal for detecting parasite DNA. Adult worm genomic DNA (1 ng) was used as a positive control sample for detecting parasite DNA (*Hha*I repeat sequence) in a Q-PCR assay. Water was used as a negative control in a PCR. For the detection of *Wolbachia *16S rDNA, sequence specific primers were employed and the PCR products were analysed by gel electrophoresis [29].

### Quantitation of interleukin and tumor necrosis factor

The secretion of interleukin-6 (IL-6) and TNFα from granulomas was measured by bioassays. For this purpose, 3 granulomas from 3 infected jirds were placed in one ml of complete medium (RPMI-1640 containing 100 U/ml penicillin, 100 μg/ml streptomycin, 1% amphotericin-B solution, and 5% fetal calf serum). The granulomas were cultured with or without 10 μg/ml *B. pahangi *adult worm soluble extract in complete medium at 37°C in 5% CO_2 _and 95% air. All culture supernatants (~200 μl) were collected from each sample at 6, 24, and 48 h; centrifuged for 10 min at 1000 RPM; and supernatants were stored at -20°C until used.

The concentrations of secreted IL-6 were determined using the well-established B9 bioassay [30,31]. In this assay, the proliferation rate of IL-6-dependent murine B9 hybridoma cells is determined after the cells are incubated with supernatants from granuloma cultures. Because the B9 bioassay is highly sensitive, IL-6 concentrations as low as 10 pg/ml were detected. Briefly, to assay IL-6 activity, supernatants from the granuloma cultures were thawed, vortexed, and added in duplicate to 96-well plates in 100-μl amounts. In parallel, equal volumes of supernatants from Con-A-stimulated jird lymph node cells [24] in duplicate were plated into 96-well plates as positive controls. An equal amount of culture medium in duplicate was included as a negative control. Thoroughly washed B9 cells were resuspended in complete medium and added to each well at a density of 2.5× 10^3 ^cells/well, and the plates were incubated at 37°C for 6, 24, and 48 h. After the cells were incubated, they were pulsed with [^3^H]thymidine (DuPont NEN Research Products, Boston, MA) for 24 h. The cells were harvested on glass-fibre filter mats with a cell harvester (TOMTEC, Hamden, CT) and counted in a Betaplate liquid scintillation counter (LKB-1205, Gaithersburg, MD). Results were expressed as net counts per minute [24].

To assay TNFα concentrations, granuloma culture supernatants in parallel with human-TNFα standards were plated into 96-well plates [32,33]. WEHI-164 murine fibrosarcoma cell line-clone 13 (American Type Culture Collection, Manassas, VA) was selected to test for TNFα cytotoxicity. Cells from stock cultures with RPMI-1640 containing a mixture of 100 U/ml penicillin, 100 μg/ml streptomycin, and 1% amphotericin-B solution were thoroughly washed and resuspended in complete medium containing 2 μg/ml actinomycin-D (Sigma Chemical co., St. Louis, MO). These cells were added to each well at a density of 2 × 10^4 ^cells/well, and the plates were incubated at 37°C for 6, 24, and 48 h with or without supernatants from granuloma cultures. Human TNFα (R & D Systems, Minneapolis, MN) in 5-fold dilutions at final concentrations of 0.002-7.39 U/ml was added in duplicate to wells containing cells. These cultures served as positive controls to generate values for a standard curve. Supernatants from the Con-A-stimulated jird lymph-node cells [24] and culture medium were also used in this assay as positive and negative controls, respectively. The cytotoxic effect of TNFα on WEHI cells cultured with or without granuloma supernatants and positive and negative controls was measured by assessing the cell viability in a methylthiazoletetrazolium (MTT) assay [34]. Subsequently, a standard curve of absorbance versus the TNFα concentration was plotted, and TNFα bioactivity in the supernatants was interpolated from the standard curve and expressed in U/ml. The minimum sensitivity of this assay to TNFα by this assay was 0.002 U/ml.

### Statistics

Analysis of variance (ANOVA) was carried out to determine whether the granulomas showed significantly different cytokine mRNA profiles. The differences were considered statistically significant at p < 0.05.

## Results

**Figure 2:**
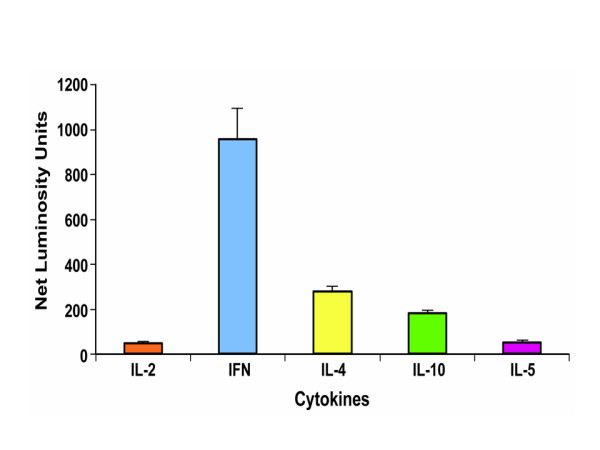
Cytokine mRNA levels in the granulomas of *B. pahangi*-infected jirds. Granulomas were obtained from the peritoneal cavity and their cytokine as well as HPRT mRNA expression levels were measured using Q-PCR. Cytokine mRNA levels are expressed as mean luminosity units ± standard error after normalizing their values with HPRT values. IL, interleukin; IFN-γ, interferon gamma.

Analysis of cytokine mRNA as determined by net luminosity units showed differences in the T-cell cytokine profile in granulomas induced by *B. pahangi *infections in jirds. In particular, the expression levels of IFN-γ mRNA were significantly (p < 0.05) higher than those of other cytokines in all granulomas from chronically infected jirds (Fig. 2). In addition, the expression of IL-4 mRNA was lower than that of IFN-γ mRNA but significantly higher than that of other cytokines (p < 0.05). The expression of IL-10 mRNA was lower than that of IL-4 mRNA but was significantly higher than that of IL-2 and IL-5 mRNA (p < 0.05). The expression levels of both IL-5 and IL-2 mRNA were significantly lower than those of all the other cytokines (p < 0.05) (Fig. 2). In summary, the granulomas contained detectable levels of the Th2 cytokines (IL-4, IL-5, IL-10, and IL-2), but the expression pattern of the Th1-type cytokine IFN-γ differed in the granulomas (Fig. 2).

**Figure 3:**
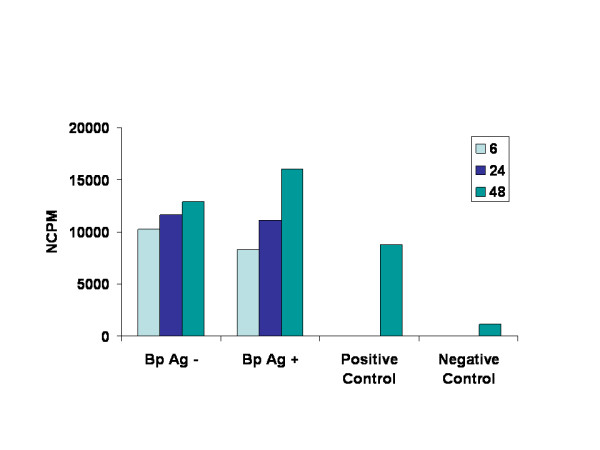
Levels of pro-inflammatory cytokine IL-6 released by granulomas. Granulomas were cultured *in vitro *for 6, 24, and 48 h with or without *B. pahangi *worm antigen. Cytokine levels were then measured in the supernatants of granuloma cultures using a B9-cell culture assay for IL-6. Results are expressed as counts per minute of [^3^H]thymidine incorporated, indicating the effect of granuloma-culture supernatants containing IL-6 on B9 cell growth. Supernatants from jird lymph node cells stimulated with Con-A and culture medium served as positive and negative controls.

**Figure 4:**
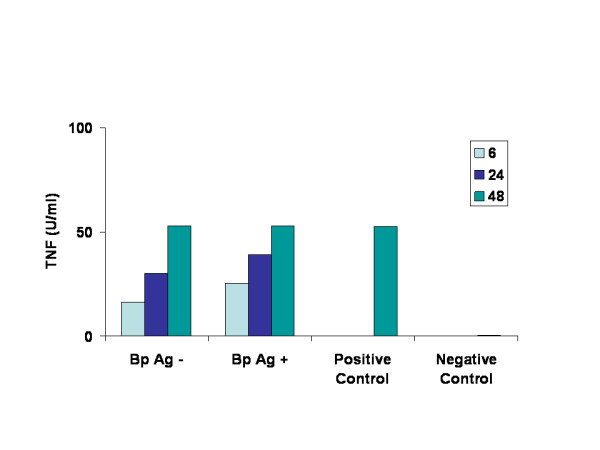
Levels of pro-inflammatory cytokine TNFα released by granulomas. Granulomas were cultured *in vitro *for 6, 24, and 48 h with or without *B. pahangi *worm antigen and secreted TNFα levels were measured in the supernatants using a WEHI-cell culture assay. Results are expressed as U/ml. Supernatants from jird lymph node cells stimulated with Con-A and culture medium served as positive and negative controls.

The granulomas spontaneously produced the pro-inflammatory cytokines IL-6 and TNF in culture (Figs. 3 &4), and under these culture conditions, the levels of these cytokines progressively increased between 6 and 48 h. From the IL-6-dependent B9 cell culture assay, we found that granuloma supernatants released IL-6 *in-vitro *and the results were comparable to those in cultures supplemented with supernatants from Con-A stimulated cells (Fig. 3). The counts per minute used to judge the stimulation of B9 cell growth indicated an increase in the granuloma secretion of IL-6 over time, but the results are statistically insignificant. Upon stimulation with adult worm antigen, granulomas released moderately high levels of IL-6. The increase in IL-6 over time upon antigen stimulation is statistically significant (p < 0.05). In parallel, the relative TNFα levels were significantly increased between 6 and 48 h (unstimulated cultures: 69% increase, p ≤ 0.05; antigen-stimulated cultures: 52% increase, p ≤ 0.05). However, no statistically significant difference was observed in TNFα levels between these two types (with and without antigen stimulation) of cultures (Fig. 4).

**Figure 5:**
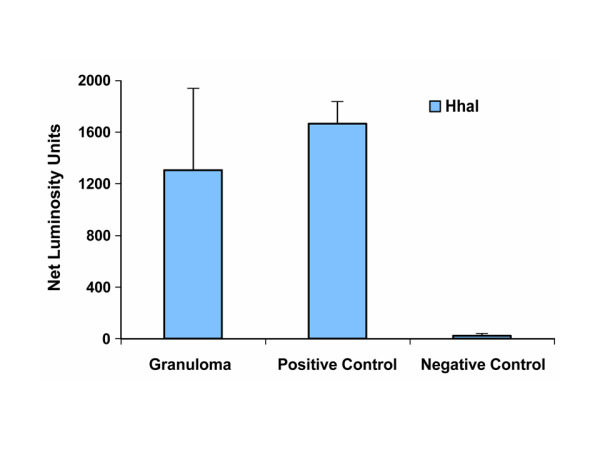
Detection of parasite DNA in granulomas by *Hha*I PCR assay. Amplified *Hha*I repeat DNA was measured in granulomas and expressed as mean luminosity units ± SD. Genomic DNA sample of *B. pahangi *adult worms served as positive control sample in *Hha*I repeat PCR assay by Q-PCR. Water was used as a negative control.

The normalized (with the background) luminosity units of Q-PCR showed the presence of parasite DNA in all granulomas. Q-PCR detected amplified *Hha*I DNA in all granulomas, and the units obtained were similar to those from adult worm genomic DNA (positive control). This confirms the presence of parasite DNA in the genomic DNA extracts of the granulomas (Fig. 5). However, the relative luminosity units of the amplified *Hha*I DNA within the granulomas varied, indicating that they may have different levels of parasites. PCR and gel electrophoresis results also showed positive signals for *Wolbachia *DNA in granulomas.

## Discussion

In our study, the Th1-type cytokine was dominant in peritoneal granulomas. Interestingly, Th1 cytokines, such as IFN-γ, are associated with inflammatory processes such as the granulomatous lesions caused by bacteria and helminthes. Similarly, our results suggest that IFN-γ plays a role in the formation of LT in subcutaneously infected jirds and in the formation of peritoneal granulomas. In subcutaneously infected jirds, IFN-γ mRNA (Th1 type) has been induced in lymphoid tissues, but in significantly lower amounts than IL-4 mRNA (Th2 type) [25,28]. In the peritoneal exudate cells of the animals whose cell populations were enriched with macrophages and eosinophils, a moderate increase in the levels of IFN-γ by 56 DAI was evident [25]. Furthermore, an increase in IFN-γ expression was also observed in spermatic cord lymphatics containing LT, but the increase was much lower than that of the other Th2-type cytokines [35]. In contrast, we also observed in the peritoneal exudate cells from jirds with i.p. infections that the IFN-γ levels were lower than those of IL-4 and IL-5 [25,26]. When jirds were infected with *Brucella *(a known IFN-γ inducer) and then with *B. pahangi *(i.p) L3, there was no increase in PGRN response to worm antigen, although *Brucella *induced high levels of IFN-γ [25]. This suggests that, although the kinetics of LT formation in lymphatics, granuloma formation in the peritoneal cavity, and PGRN responses in jirds are similar, the immunological modulators involved in the lymphoid and nonlymphoid tissues differ. It is also possible that IFN-γ plays a key role in the induction of one or all these multiple responses, but it is unlikely that IFN-γ is the only cytokine contributing to the formation of granulomatous lesions; it may simply have a small role in the complex interaction of Th1 molecules with those of Th2 type.

Filariae in general are thought to develop a polarized Th2-type host immune response and have a severely impaired ability to produce Th1-type cytokines in humans and rodents. The development of the hyporesponsive chronic disease state in humans appears to be complex, involving Th1 and Th2 cytokines and other mechanisms of innate immunity [36-39]. Moreover, it has been shown that immune effector mechanisms involved in hyporesponsive microfilaremic states also involve an antagonistic Th3/Th1-type response [40].

Previous studies indicated that the renal lymph node (the lymphoid tissue draining the infected lymphatics) has the highest levels of IL-10 mRNA during the chronic phase of infection [25,35]. However, proportional increases in IL-10 similar to those seen in the spleen and peritoneal exudate cells were not observed in granulomas. Similar studies conducted in jirds infected i.p. with *B. pahangi *also failed to show a distinct role for IL-10 in the down-regulation of the PGRN response. It is possible that IL-10 acts at the site of infection to down-regulate LT formation, and it is unlikely that granuloma formation in the peritoneal cavity is mediated by IL-10 in infected jirds. The expression profile of cytokines indicate that peritoneal exudate cells [25] may assist in forming the granulomas further supports this hypothesis.

IL-5 mRNA expression is transitory in the *Brugia*-jird model. The peak in peripheral eosinophilia at 14–28 DAI reflects the peak of IL-5 mRNA expression observed in the lymphoid tissues of infected jirds [25,28,41]. In subcutaneously infected jirds, this eosinophilia peaks at approximately 28 DAI, and IL-5 mRNA levels increase between 14 and 28 DAI, indicating a role for the systemic IL-5 response in infected animals. Levels of IL-5 mRNA were low in all tissues in chronically infected jirds. We observed that the levels of IL-5 tend to be low in worm granulomas. Eosinophilic granulomas in *Wuchereria *infections were observed earlier [42]. In mice and jirds, granulomas contained eosinophils close to the worms, but their numbers were significantly lower than in macrophages and giant cells [18,22], suggesting that IL-5 does not play a significant role in the development of granulomas at the chronic stage, unlike it does in these other cell types involved in phagocytosis. In addition, the profile of IL-5 mRNA expression, which differs from that of IFN-γ, suggests that these two cytokines play different roles in the formation of granulomas. Moreover, IL-5 was found to play a major role in the recruitment of neutrophils to the site of *L. sigmodontis *infections in BALB/cByJ mice and in the development of nodules [43] in which macrophages and eosinophils were the predominant cell types. More recently, a synergism between the Th1 (IFN-γ) and Th2 (IL-5) cytokines has been observed in murine filariasis, leading to the containment of infections [44]. The expression profiles of these cytokines in human filarial granulomas remain to be determined. Interestingly, in human mycobacterial infections, it has become evident that bacteria and host T cells collaborate in granuloma formation, and some nonspecific T-cell subsets play a role in granuloma formation [19,20]. It is unclear whether this is the case in filarial granulomas.

The spontaneous release of the inflammatory cytokines IL-6 and TNFα strongly supports their role in inducing inflammation in the peritoneal cavity and possibly in lymphatics. It is likely that, in the peritoneal cavity, these cytokines are responsible for recruiting inflammatory cells and other mediators causing tissue necrosis and the deposition of collagenous material around tissues of parasite origin.

On the basis of the morphology and development of granulomas and nodules, it has been speculated that filarial granuloma development reflects an innate immune mechanism by which incoming larvae, developing larvae, microfilariae, and adult worms are eliminated [18,43,45]. It is possible that filaria infected hosts (for example, rodents and humans) clear the active infections, such as granulomas in mycobacterial infections and schistosomiasis, by developing granulomas [18,46,47]. If granuloma development is a means of containing the live or dead parasite, leading to its elimination from the body, then understanding how cytokines and chemokines are involved in this process might give us clues in the search for suitable therapeutics to contain worm development and the subsequent granulomatous inflammation associated with filarial infections.

Interestingly, a reexamination of transmission electron microscopy photomicrographs of granulomas [22] revealed intact and degenerating worm fragments containing *Wolbachia *(data not shown). PCR assays using genomic DNA and *Wolbachia *specific primers for 16S rDNA confirmed the presence of bacterial components in these granulomas [29]. Therefore, it is likely that inflammatory responses leading to granuloma development are complex and might be triggered by parasite proteins or bacterial components, or both. Additional studies are needed to understand the interaction of *Wolbachia *in the granuloma formation.

Although the jird-*B. pahangi *model of human lymphatic filariasis has been useful for understanding the immunological events associated with active infections, there has been no new information about the roles of individual immune effector molecules in granuloma development as the infection progresses. We recently investigated the expression of various classic Th1 and Th2 cytokines following L3 infections of *Brugia *in the jird model [28,35]. The results strongly suggest that much of the cellular response of the granulomas developed in the peritoneal cavity is immune mediated, differs from the cytokine expression in lymphoid tissues, and also reflects what happens in infected lymphatics. A more detailed study of cytokine profiles in lymphatic granulomas developed soon after infection, at the acute stage, and at the chronic stage would further define the role of granulomas in the pathogenesis of lymphatic filariasis.

## Competing interests

The author(s) declare that they have no competing interests.

## Authors' contributions

TRK assisted with the *in vivo *and *in vitro *experiments and made recommendations on the experiments. RUR conceived the study, performed the *in vivo *and *in vitro *experiments, interpreted the results, and drafted the manuscript. All authors approved the manuscript for submission to *Filaria Journal*.

## Financial support

The work presented in this manuscript was supported by National Institutes of Health grant AI-19199-18.
